# Ancestral Stories of Ghanaian Bimoba Reflect Millennia-Old Genetic Lineages

**DOI:** 10.1371/journal.pone.0065690

**Published:** 2013-06-12

**Authors:** Hernando Sanchez-Faddeev, Jeroen Pijpe, David van Bodegom, Tom van der Hulle, Kristiaan J. van der Gaag, Ulrika K. Eriksson, Thomas Spear, Rudi G. J. Westendorp, Peter de Knijff

**Affiliations:** 1 Department of Human Genetics, Leiden University Medical Center, Leiden, The Netherlands; 2 Leyden Academy on Vitality and Ageing, Leiden, The Netherlands; 3 Department of Gerontology and Geriatrics, Leiden University Medical Center, Leiden, The Netherlands; 4 Department of History, University of Wisconsin-Madison, Madison, Wisconsin, United States of America; University of Utah, United States of America

## Abstract

Oral history and oral genealogies are mechanisms of collective memory and a main cultural heritage of many populations without a writing system. In the effort to analytically address the correspondence between genetic data and historical genealogies, anthropologists hypothesised that genealogies evolve through time, ultimately containing three parts: literal – where the most recent ancestry is truthfully represented; intended – where ancestry is inferred and reflects political relations among groups; and mythical – that does not represent current social reality. While numerous studies discuss oral genealogies, to our knowledge no genetic studies have been able to investigate to what extent genetic relatedness corresponds to the literal and intended parts of oral genealogies. We report on the correspondence between genetic data and oral genealogies among Bimoba males in a single village in North-Eastern Ghana. We compared the pairwise mismatch distribution of Y chromosome short tandem repeat (Y-STR) haplotypes among all lineages present in this village to the self-reported (oral) relatedness. We found that Bimoba are able to correctly identify unrelated individuals in 92% of the cases. In contrast, they are able to correctly identify related individuals only in 38% of the cases, which can be explained by three processes: (1) the compression of genealogies, leading to increasing inaccuracy with increasing genealogical distance, (2) inclusions into the lineage from intended relations such as clan co-option or adoptions, and (3) false paternities, which in this study were found to have a minor effect on the correspondence between genetic data and oral genealogies. In addition, we observed that 70% of unrelated pairs have from six to eight Y-STR differences, a diversification peak which we attribute to an ancient West African expansion dating around 9454 years ago. We conclude that, despite all caveats, oral genealogies are reflecting ancient lineages more accurately than previously thought.

## Introduction

Anthropologists and historians have long debated the historical validity of oral genealogies. There is a general consensus, however, that genealogies function as charters for social groups, with each ancestral node seen as the progenitor of a particular clan or lineage [1]–[3]. Over time, ancestors who did not give rise to such groups are dropped from the genealogy through an on-going process of ‘structural amnesia’, leading to telescoping of the genealogy into a stable number of generations [1]–[7]. In the process, genealogies assume a familiar tripartite form, including 1) a mythic period of origins in which major events, occurring over centuries, are compressed into a single generation, 2) a subsequent period of repetitive social processes of lineage segmentation extending over several generations, and 3) a final era of the remembered history of discrete individuals and events over the last three to four generations [8]. Thus, the historical form and meaning of different generations may differ sharply from one another [8]–[10]. At the same time, however, genealogies can be formed by the addition of individuals and groups as a result of intended relations, such as co-optation of other lines, conquest and incorporation of neighbours, or outright fabrication, to reflect current social reality [2]. Thus, while historians and anthropologists generally agree that genealogies can rarely be used to establish an absolute historical chronology, they may still accurately represent social reality on the ground [11]. In the absence of other sources, oral genealogies are the single source of historical information of a group of people, and the question of its use and reliability remains important. We initiated a genetic study to investigate to what extent oral genealogies can be used to infer historical relationships, by comparing oral genealogies with genetic relatedness in a single village of the Bimoba, an ethnic group in North-Eastern Ghana.

The Bimoba lifestyle is in many cultural and social aspects typical of the rural Sahel region [12], [13]. Bimoba live under adverse conditions. Income is mainly generated through subsistence agriculture and varies from year to year [14]. The notion of ancestral spirits endorses the maintenance of oral genealogies beyond what is customary in most Western societies [12]. Bimoba often request ancestral spirits for aid and assistance in many matters of life and invest into supporting special requests through animal sacrifice. Animal sacrifice is performed at a special hut dedicated to their ancestors, an integral part of each Bimoba compound. These compounds are inhabited by an extended family consisting of a landlord, his wives and children and often the landlord’s younger brothers. Several extended families form a clan. The clan is an important factor in the familial social support system. Within clans, land and goods are inherited patrilineally [12]. Bimoba society is patrilocal, and marriage practices include polygyny and clan-exogamy [15], *i.e.* the prohibition to marry within the clan. The ancestry of men is rigorously kept and eventually becomes part of clan’s oral history. Within each clan, men claim common descent from a male progenitor that is one of the three mythical founders of the ethnic group Bimoba. Women join their husband’s clan upon marriage and their ancestry is not preserved. For men, there are two possibilities to switch clan membership, both of which give an example of intended relations. In the rare case of adoption, the child loses the clan of his biological father and becomes part of the clan of his stepfather. Politically motivated cases of clan switching are also known, but not widespread. A single such case has been reported in the village of study in the present generation. Further evidence for the importance of clans comes from a previous genetic study in which we showed that the distribution of Y-chromosomal variation in the village established boundaries that corresponded closely with the clan structure [16].

We collected and organized the oral history from a single Bimoba village into oral genealogies by assessing the composition of each household in the village, and by interviewing the village elders about relationships among households and among clans. To evaluate the correspondence of these oral genealogies with genetic relatedness, we have performed genetic testing. Y-chromosome Short Tandem Repeats (Y-STRs) are particularly well suited for this genealogical DNA testing [17]. Since these markers are located on the Y-chromosome, they are transmitted from father to son only (uniparental). Occasionally during generations of transmission, mutations occur that give a son one tandem repeat difference with his father. The appearance of a mutation is a function of the number of generations. Therefore closely related males have similar Y-STR profiles, whereas distantly related males are more likely to have multiple differences in their Y-STR profiles. We calculated the expected degree of relatedness for all pairs of individuals in the genealogies by scoring the number of repeat differences in 15 Y-STR loci [18] and compared the results to the self-reported relatedness.

## Results

**Figure 1:**
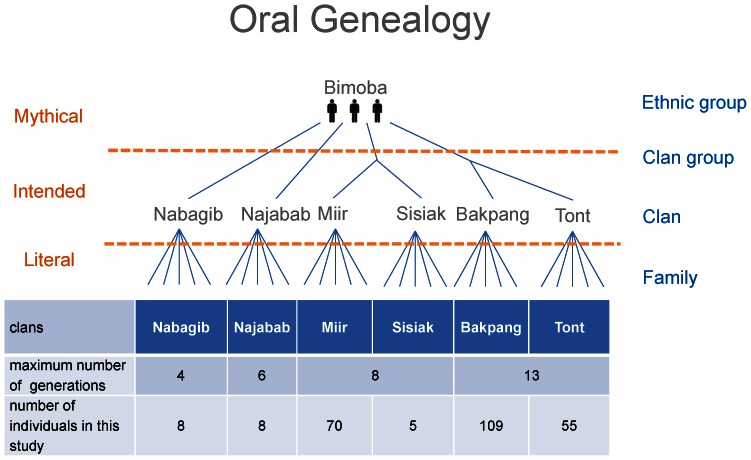
Oral genealogies of a Bimoba village. A schematic model of the collected genealogies and the social structure of 255 Bimoba men, with a summary of their genealogical and genetic data. The theoretical anthropologic classification of oral genealogies is depicted on the left. Historians and anthropologists distinct three levels in oral genealogies: a literal level that represents the immediate past, an intended level that contains information of the social relations, and a mythical level where a direct relation is unclear. The family units are organized into six clans. Two pairs of clans are joined by a reported common male ancestor.

We reconstructed self-reported oral genealogies of all the Bimoba men from a single village in North-Eastern Ghana and collected saliva swabs of 255 males across all households. The oral histories were assembled into genealogical trees. The genealogies contain males that are up to 26 meiosis events apart, *i.e.* with a maximum Time to the Most Recent Common Ancestor (TMRCA) of 13 generations. All six Bimoba clans present in this village agreed to participate. We combined the genealogies of two pairs of clans that are reported to share a distant mythical Bimoba ancestor, resulting in four clan-groups (Figure 1). However, since mythical ancestors are known not to correspond to the reality on the ground [11], [19], the relation to the mythical ancestor was not taken into account when analyzing the correspondence between genealogical and genetic data.

**Figure 2:**
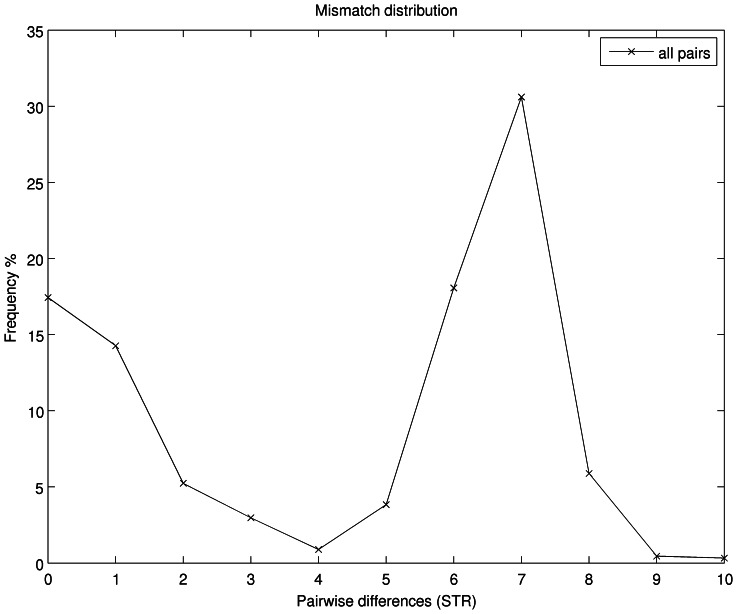
Mismatch distribution of Y-STR haplotypes in a Bimoba village. The mismatch distribution of the number of Y-STR differences in all pairs of 255 men, expressed as the percentage of observed pairs (32385 pairs). A higher number of Y-STR differences indicates a longer time to the most recent common ancestor.

**Figure 3:**
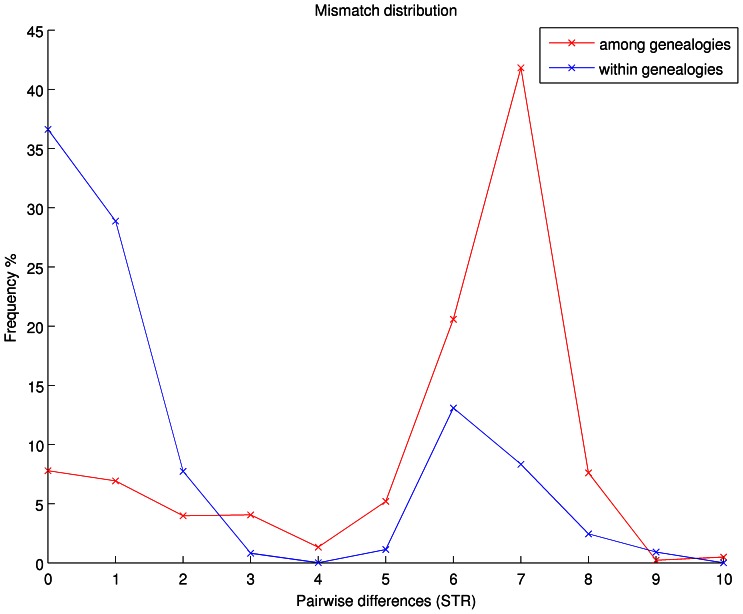
Mismatch distribution within vs. among reported genealogies. The mismatch distribution of the number of Y-STR differences, expressed as the percentage of observed pairs among genealogies (red line; 21589 pairs) or pairs within genealogies (blue line; 10796 pairs).

We present the genetic data as a mismatch distribution of the number of Y-STR differences for all pairs of males in our dataset (Figure 2). We observed a bimodal distribution with a peak at zero differences and a peak at seven repeat differences. We hypothesised that the first peak represents male pairs related within genealogies, *i.e.* closely related pairs, and the second peak represents male pairs from different genealogies, *i.e.* distantly related pairs. To further address this hypothesis we separately present the mismatch distributions for pairs among genealogies, *i.e.* unrelated males, and within genealogies, *i.e.* related males (Figure 3).

### Among Genealogy Analyses

The mismatch distribution of unrelated males (Figure 3: red line) illustrates the relatedness among genealogies. The ability of Bimoba to identify differences among distantly related lineages is a specificity test. In the case of oral genealogies we define specificity as the ratio of pairs that have at least one Y-STR difference over all the possible unrelated pairs. From all the pairs that are reported to be from different lineages, 92.2% have at least one Y-STR difference. This corresponds to a genetic distance equivalent to at least 19 meiotic events, as calculated with a cumulative mutation rate obtained from Ballantyne et al. [20], (Table S1). The remaining 7.8% of the pairs share identical Y-STR haplotypes despite being reported to be unrelated. This analysis establishes a lower bound of 92.2% for the specificity of oral genealogies, *i.e.* for the ability of oral genealogies to differentiate between men from different genetic lineages.

In the distribution of unrelated males, 70% of pairs have six to eight Y-STR differences in,a peak in the red line on Figure 3. Harpending suggested that such peaks represent a population expansion [21]. Slatkin and Hudson extended this suggestion, showing that population expansion forces coalescent events into a narrow time window [22]. They explained that peaks are representative of either on-going expansion, past expansion or selection pressure effects. Since the markers employed here are specifically selected for neutrality and selection is unlikely, we assumed that the subjects of our study experienced a demographic expansion. Slatkin and Hudson indicated that the exact position of the peak does not correspond to the start of the expansion per se, but that it appears during an expansion event. The distance of seven Y-STR differences at which the peak is located suggests that such an expansion was unlikely to be recent. Assuming an exponential expansion, we estimated the TMRCA of the males in this village to 221 generations (95% CI: 206–238 generations). With a generation time of 42.78 years, obtained through demographical assessment of 13866 father-son pairs in this and neighbouring villages, the above generation time corresponds to 9454 years ago (ya) (95%CI: 8813–10182 ya).

### Within Genealogy Analyses

Next, we employed a sensitivity test to address the ability of the Bimoba to identify relationships *within* their genealogy. Here sensitivity is the percentage of pairs for whom the observed number of Y-STR differences corresponds to the expected number of Y-STR differences. We established the expected amount of Y-STR differences by repeatedly simulating the Y-STR transmission events, assuming a correctly reported genealogy. We employed a step wise mutation model [23] with locus specific mutation rates reported by Ballantyne et al [20] (Table S1). From these simulations we expected to find almost all (98.8±2.52% s.d.) of the pairwise comparisons to have no Y-STR differences. We observed only 36.6% of all pairs to have an identical haplotype (Figure 3: blue line – zero differences). We expected to find 1.2±2.5% of all pairwise comparisons to have one Y-STR difference, whereas we observed 28.8%. In contrast with the observed number of pairs we did not expect to find any pairs to have two or more Y-STR differences. This results in a sensitivity of 37.8% (36.6% +1.2%) of all the observed pairs that are related as expected from the simulation. Judging from the mismatch distribution (Figure 3: blue line), reportedly related pairs that have at least one Y-STR difference fall into two discrete categories: 26.9% have five or more differences and are therefore genetically very distant from each other, and 36.6% of all pairs have one to three Y-STR differences. Further analysis revealed that each individual in a pair with five or more Y-STR differences (26.9%) belonged to a separate subgroup of the Y-E1b1a*(M2) haplogroup [16]. Each subgroup diverged from the M2 branch several thousand years ago [24], [25]. We therefore refer to such pairs as unrelated. The 36.6% of all pairs with one to three Y-STR differences belong to the same subgroup of the Y-E1b1a*(M2) haplogroup. These pairs are therefore related but more distantly related than reported. To further investigate the relationship between the number of meiosis events and the number of Y-STR differences, we visualised the changes in the mismatch distribution by starting with father-son pairs and consecutively adding more distant relations (Figure S1, Movie S1). For fathers and sons we observe more pairs of zero Y-STR differences than for more distantly related pairs. This demonstrates a better awareness of the relatedness of fathers and sons than of more distant relationships.

### False Paternity

The 15 Y-STR loci used have an independently estimated cumulative mutation rate that, on average, produces one random repeat difference in 19 meiosis events (cumulative mutation rate of the markers) [20]. Therefore, our method reliably measures the differentiation of distantly related pairs (among genealogies) but has a limited resolution for closely related pairs within a genealogy, such as parental or grandparental relationships, where no Y-STR differences are expected. To meet the uncertainty that rises from a limited resolution for pairs within a genealogy, we estimated the accuracy of the genealogical data of the extant generations by additional analyses of autosomal microsatellites. Our analyses revealed eight unmatched father-son pairs out of 116 (6.9%). To address sampling errors as a source, we additionally analysed 27 mothers. We found one trio where the mother did not match the son (3.7%). The observed false paternity rate in the trios is therefore 6.9±3.7%. To estimate the influence of false paternities in the current generation on our mismatch distributions, we reanalysed the Y-STR data after correcting for the eight detected false paternities by replacing the mismatched Y-STR haplotypes with haplotypes matching the lineage. The correction resulted in a minor change, with an average of 0.13% per each position in the mismatch distribution plot with a maximum change of 0.5% at distances of one and seven Y-STR differences (not shown due to overlapping lines). This result shows that the error introduced by false paternities in the extant generations alone does not explain the deviation from the expected number of Y-STR distances.

## Discussion

We studied the accuracy of genealogies in a society with an oral history tradition by comparing genealogical distances to genetic distances in genealogies of men from a single village. We detected a bimodal mismatch distribution of the genetic distances of the males in this village, which we separated into two mismatch distributions: among genealogies (specificity) and within genealogies (sensitivity). We have found that genealogical specificity is higher than genealogical sensitivity. In other words, individuals are better able to differentiate unrelated individuals from another clan than to correctly asses a relationship within the familial or clan genealogy. The specificity of 92.2% is unexpectedly good, allowing follow-up population genetics studies to rely on oral information to collect samples from unrelated individuals.

On the other hand, the ability to correctly identify related individuals is less than we anticipated. For father-son pairs this ability is good (93.1%±3.7%), but it drops fast with longer genealogical distances. The low sensitivity of oral genealogies is due to two effects: 1) There is a markedly higher than expected frequency of pairs with one to three Y-STR differences within genealogies (Figure 3 - red line), and 2) the moderate but unexpected amount of more distantly related males within genealogies. Initially, we suspected this to be due to a large amount of genealogical errors, such as the accumulation of the effect of false paternities. However, when we corrected for the few false paternities detected by autosomal STR analysis in the extant generations, the change was minor. Moreover, if false paternities alone would be responsible for the low specificity, in this small village it should have produced a high number of closely related pairs among reportedly unrelated men as well. We observe that only 7.8% of pairs among lineages have no Y-STR differences. The percentage of such pairs among genealogies is in the same range as the 6.9%±3.7% false paternities detected by autosomal STR analyses. We suggest that these closely related pairs among genealogies correspond closely to the true error due to false paternities in this population. Because the influence of false paternities appears to be limited, the remaining higher than expected frequency of pairs within genealogies that have one to three Y-STR differences points to compression and telescoping of these genealogies [1]–[8], *i.e.* the omission of progenitors in the memory of the extant generations. The unexpectedly high frequency of pairs that have five or more Y-STR differences indicates a substantial amount of distant relationships within clans. We speculate that the high number of pairs in this part of the distribution is due to cumulative effect of false paternities over many generations and intended relations such as clan co-option or adoptions [2]. A single case of clan co-option, a clan-switching in a extant generation, was reported in the village.

We found that among the Bimoba in this village a high frequency of pairs show seven Y-STR differences. We provisionally attributed this diversity to an ancient demographic expansion in West Africa about 221 generation ago. This has similar dating as the hypothesised Bantu expansion in Africa, which recently received considerable attention [25], [26]. Ansari Pour et al. estimated the TMRCA of the E1b1a Y-haplogroup (the dominant African Y-haplogroup) using 43 African population samples to be 255 generations [26]. In another study by Montano et al. among populations in Nigeria, Cameroon, Gabon and Congo the expansion of Bantu languages was dated [27] to 200 generations ago [28]. However, both Montano et al. and Ansari Pour et al. used a generation time of 25 years. This leads to a different timing of an expansion in Africa, 5000ya and 6382ya, respectively, versus 9454ya in the present study. A correct generation time is crucial for fine tuning the various estimates. The generation time of uniparental markers, markers inherited by both genders separately (Y-chromosome and mtDNA), is an average age of the parents when producing their offspring [29], which in Africa and elsewhere is different for males and females [29]. In traditional African societies the age of marriage of women is often much lower than the average age of marriage of men [30], leading to drastically different generation times of sexes. In order to estimate the correct age of the father we have analysed the age of the father at child birth in 13866 father-child pairs in this and other neighbouring villages, resulting in an average age of 42.78 years. Using such age as generation time resulted in TMRCA of 9454ya. Unlike the male generation time, in Sub-Saharan Africa the generation time of 25 years (or even lower in the Ghanaian study region) is better suited for generation time estimate of mtDNA markers, markers inherited uniparentally by women. Estimates from mtDNA by Barbieri et al. [31] in neighbouring Burkina Faso of an ancient demographic expansion using female generation time of 25 years led to a dating, from 9000 to 14000 ya. This coincides with our estimate of Y-STR data in males, suggesting that we observe a related expansion event in our Ghanaian data. Barbieri et al. point out that such dating overlaps with the Holocene Optimum and therefore the end of Younger Dryas, 10000–11000ya [32]. During the Younger Dryas the aridity and consequently the vegetation radically changed in Africa [32]. Following the Younger Dryas, the Holocene climate optimum favoured the spread of agriculture and colonization of new territories which is in turn associated with a demographic expansion of lineages in many African populations [33]–[35].

In conclusion, we find that in a patrilocal and patrilineal society, genealogies inferred from oral history are suited to identify unrelated lineages. In reportedly related lineages, the reduced sensitivity can be explained by three processes: 1) the telescoping of these lineages, 2) intended relations from clan co-option and adoptions, and 3) false paternity. The number of pairs with one and two Y-STR differences is much higher than expected, indicating that the genealogies of the elder men we interviewed have two to three times more ancestors than reported. This result suggests that telescoping is an integral part of the Bimoba’s oral tradition. Further genetic studies are required to confirm the ability of oral genealogies to distinguish related from unrelated group members and to establish the generality of telescoping in oral genealogies.

## Methods

### Study Setting

The Bimoba (or Moba in Togo) constitute approximately 370,000 individuals of which approximately 50,000 live in North-Eastern Ghana and the remaining part in adjacent areas in Togo. The study village Farfar is located in the Upper East Region of Ghana, at 10°44′23.41′′N and 0° 9′43.69′′W. The sampling was done in the framework of the Garu Health & Life Research project of Leiden University Medical Center, department of Gerontology and Geriatrics. The Bimoba in Farfar are traditional agriculturalists that produce food at subsistence level. The population has a patrilocal and patrilineal structure: the women are accepted to their husbands’ clan, sons stay in or near their fathers’ compound. Polygyny is widespread and households are typically large. Detailed ethnological information can be found in Meij 2008 [12].

### Ethics Statement

Informed consent of each participant for this study was obtained through a local field staff translator. Because of low levels of literacy in the research area, informed consent was obtained orally from the participants in their own language. Participation only proceeded after verbal consent of the participant. The consent procedure and the study were approved by the Medical Ethical Committee of Leiden University Medical Center and the Ghana Health Service Ethical Review Committee.

### Genealogical Analysis

Genealogical data was collected from interviews during field visits to each household. The interviews were taken by a staff member from The Netherlands together with a translator, a member from the local field staff enrolled in the Garu Health & Life Research project. The translator was a lifelong inhabitant of the village under study. Basic demographics of the study population; name, sex and age, were collected in 2002 [36]. During the collection of the genealogies, we used the database reported by Ziem et al. [36] and visited all the individual households of the village. The demographic information was assessed each year during the annual follow-up by revisiting all households in the village subject of study as well as in the neighbouring area of approximately 360 square kilometres, collecting information of all the inhabitants and registering individuals that were newly born, deceased, or migrated. We also performed random double visits. Double visits have shown that the database is accurate and reliable. To collect genealogies in the village, the subject of this study, we interviewed the head of the household (landlord) and other household members present on the relationship between the different household members. We stressed that we were interested in the biological relation, which we made explicit as the person that actually gave birth to a child and not the one who was taking care of a child. In addition, we specifically asked about cases of none-biological relatedness (e.g. adoptions) and excluded them from our genealogical data. The landlord reported the ethnic group and the clan of each compound inhabitant. All households in the village participated in the study, resulting in data of six different clans being collected. We asked the landlord about his relation to the other households in the village. In this way we could connect the genealogies of the households together through the male lineage. Finally, in addition to the interviews on the relationships within and across households, we interviewed the elders of the village from different clans on their male ancestors. They all concluded their ancestry with the clan founder as a direct descendant of one of the three Bimoba founders. The genealogical data was captured using Cyrillic CO software to make genealogical trees. The legendary figures mentioned by the elders were omitted from the analysisGenetic analyses.

Genetic material was collected using buccal swabs and stored in a buffer. DNA was extracted with the QIAamp DNA Mini Kit (Qiagen), according to the manufacturers’ standard protocol. A total of 255 males were included in the study, sampling wherever available at least two males per household. These men encompass 116 father-son pairs.

The AmpFℓSTR®Yfiler™ PCR Amplification Kit (Life Technologies) was used for typing 17 Y-chromosomal STR loci. DYS19, DYS389I, DYS389II, DYS390, DYS391, DYS392, DYS393, DYS385a, DYS385b, DYS437, DYS348, DYS439, DYS448, DYS456, DYS458, DYS635, and Y-GATA-H4 were genotyped according to the manufacturer instructions. The kit includes two multiple copy markers, DYS385 and DYS389. In case of DYS389, Y-filer separately amplifies DYS389A and the complete segment DYS389A+B. In order to obtain an independent value for DYS398B, we subtracted DYS389A from DYS389A+B segment. DYS385, another multiple copy marker, is also composed by two separate markers. However using Y-filer results alone it is not possible to determine the exact location. Therefore both DYS385A/B markers were omitted from the analysis, resulting in 15 loci employed. Autosomal STRs were typed by the use of forensic Powerplex®16 (Promega), AMPF*l*STR® Identifiler® (Life Technologies). PCR reactions were performed according to the manufacturers' manual specifications. PCR products were analysed using an ABI 3100 automated DNA sequencer (Life Technologies) and the Gene marker software (Softgenetics CO). The mismatch distribution analyses were implemented in Matlab CO software. The analysis routines are available upon request.

### Estimation of TMRCA

We estimated the TMRCA using Y-time [37] assuming exponential population growth and a single-step mutation model with Y-STR locus specific mutation rates obtained from Ballantyne et al [20], see Table S1. The average fathers’ age at birth was estimated using demographic data of this and neighbouring villages in an area of approximately 360 square kilometres. The age estimate employed was obtained by asking for an official identification, such as birth certificate, or in case of absence of such, the average of independent estimates of Dutch and Ghanaian fieldworkers. Out of 14434 father-child pairs interviewed, 568 were discarded due to an evaluated age difference of less than 15 years. These removed pairs correspond either to demographic database capturing errors or erroneous age estimation. Constant mutation rate and constant generation time were assumed in the model.

### Simulation of the Clan Genealogies

We estimated the expected number of Y-STR differences in the reported genealogies by simulating the transmission of mutations to the descendants and assuming that the genealogies are correct. The probability of the mutation of particular marker was established by a mutation rate from Ballantyne et al. [20] (Table S1). As in the TMRCA estimation, we assumed a single-step mutation model. Due to limited genealogy size (at most 13 generations), only cases with single mutation appeared after 10,000 simulations.

### Data Availability

Table S2 contains a full Y-STR data table. Genealogical data are available upon request with the corresponding author only.

## Supporting Information

Figure S1 **Mismatch distribution of Y-STR haplotypes with increasing distance in the genealogies.** The main figure shows a series of lines with a gradient from dark red to pale red, corresponding to the shortest possible distance between pairs of males in a pedigree (father-son pairs with one meiotic step difference) to the largest distance (very distant cousins with 25 meiotic steps different). For five specific Y-STR difference classes (0, 1, 2, 6, and 7) we show the same data by means of bar graphs. Please note that in these bar graphs the Y-axis scales differ. The percentage of male pairs with zero (0) Y-STR differences declines from 90.4% among father-son pairs to 36.6% among the most distant pairs. As is clearly shown, for the other Y-STR difference classes we see a reversed pattern.
(TIFF)

Table S1 **Mutation rates of Y-STR markers used in this study.** Mutation rates obtained from Ballantyne et al. [20]. The inverse of batwing estimates was calculated in order to obtain the probability of mutation per marker. The cumulative mutation rate is therefore 0.052, which corresponds to a probability to observe a mutation once in 1/0.052 = 19 meiosis events.
(PDF)

Table S2 **Y-STR data used in this study.**
(CSV)

Movie S1 **Frame sequence of mismatch distributions with increasing distance in the genealogy.** Each frame is a mismatch distribution of pairs with increasing number of reported relatedness. Mismatch distribution lines are sequentially added as frames of the movie. The first frame represents father-son pairs, consecutively adds more distant relations and the sequence ends with the last frame representing relations separated by 26 meiosis events (13 generations).
(MPG)
