# Supplementary figures and images for: Ancestral Stories of Ghanaian Bimoba Reflect Millennia-Old Genetic Lineages
Source: PLoS One. 2013 Jun 12;8(6):e65690. doi: 10.1371/journal.pone.0065690 (PMC3680464; doi:10.1371/journal.pone.0065690)

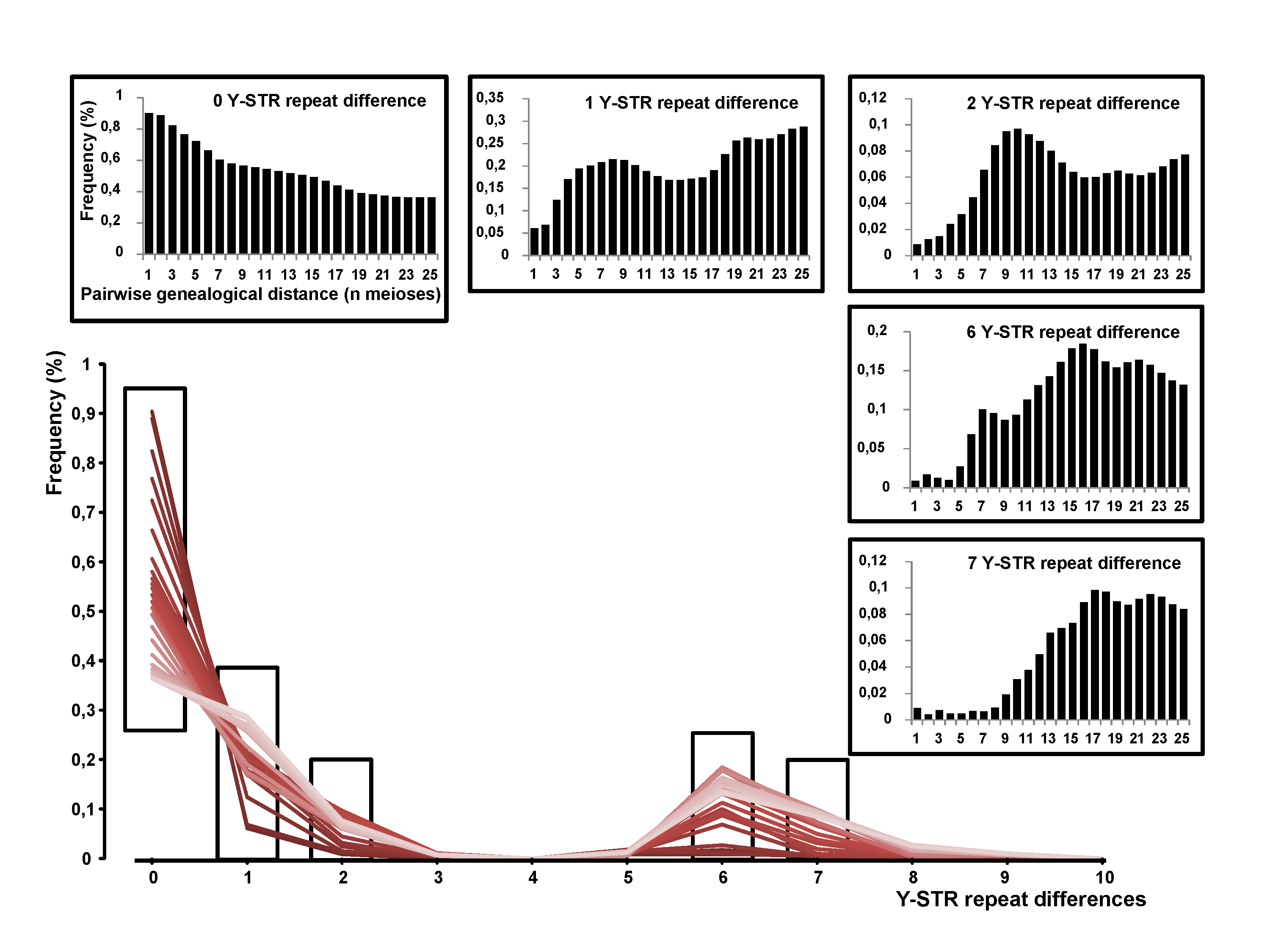

Supplement: Figure S1 — Mismatch distribution of Y-STR haplotypes with increasing distance in the genealogies. The main figure shows a series of lines with a gradient from dark red to pale red, corresponding to the shortest possible distance between pairs of males in a pedigree (father-son pairs with one meiotic step difference) to the largest distance (very distant cousins with 25 meiotic steps different). For five specific Y-STR difference classes (0, 1, 2, 6, and 7) we show the same data by means of bar graphs. Please note that in these bar graphs the Y-axis scales differ. The percentage of male pairs with zero (0) Y-STR differences declines from 90.4% among father-son pairs to 36.6% among the most distant pairs. As is clearly shown, for the other Y-STR difference classes we see a reversed pattern. (TIFF) [file pone.0065690.s001.tiff]
