# Supplementary material for: Ancestral Stories of Ghanaian Bimoba Reflect Millennia-Old Genetic Lineages
Source: PLoS One. 2013 Jun 12;8(6):e65690. doi: 10.1371/journal.pone.0065690 (PMC3680464; doi:10.1371/journal.pone.0065690)
Supplement: Table S1 — Mutation rates of Y-STR markers used in this study. Mutation rates obtained from Ballantyne et al. [20]. The inverse of batwing estimates was calculated in order to obtain the probability of mutation per marker. The cumulative mutation rate is therefore 0.052, which corresponds to a probability to observe a mutation once in 1/0.052 = 19 meiosis events. (PDF) [file pone.0065690.s002.pdf]

Mutation rates of Y-filer markers employed.

DYS456	DYS389CD	DYS390	DYS389AB	DYS458	DYS19	DYS393	DYS391	DYS439	DYS635	DYS392	GATA_H4	DYS437	DYS438
0.006429	0.003570373	0.002356	0.002543	0.01045	0.002338	0.000766	0.002694	0.006162	0.006249	0.00054	0.005279	0.001369	0.000438

Mutation rates obtained from Ballantyne, K. N. et al. Mutability of Y-Chromosomal Microsatellites: Rates, Characteristics, Molecular Bases, and Forensic Implications. The American Journal of Human Genetics 87, 341-353, doi:10.1016/j.ajhg.2010.08.006 (2010).
